# Vitamin and mineral supplements and fatigue: a prospective study

**DOI:** 10.1007/s00394-025-03615-y

**Published:** 2025-02-22

**Authors:** Sisi Xie, Pedro Marques-Vidal, Vanessa Kraege

**Affiliations:** 1 Department of Internal Medicine, Centre Hospitalier Universitaire Vaudois (CHUV), Lausanne University Hospital, Rue du Bugnon 46, 1011 Lausanne, Switzerland; 2 Medical Directorate, Lausanne University Hospital (CHUV), Lausanne, Switzerland; 3 Innovation and Clinical Research Directorate, Lausanne University Hospital (CHUV), Lausanne, Switzerland; 4 Faculty of Biology and Medicine, University of Lausanne, Lausanne, Switzerland

**Keywords:** Vitamins, Minerals, Fatigue, Epidemiology, Nutrients

## Abstract

**Purpose:**

The consumption of vitamin/mineral supplements (VMS) and vitamin/mineral and/or dietary supplements (VMDS) is popular among the general population. However, the association of VMS/VMDS with fatigue remains sparse and conclusions are mixed. We aimed to understand the association between VMS/VMDS and fatigue.

**Methods:**

Prospective study in the city of Lausanne, Switzerland, including 1361 participants (50.3% female, mean age 61.0 ± 9.4 years). Participants were divided into VMS/VMDS users and non-users. Fatigue levels were assessed using the Fatigue Severity Scale (FSS) and the 14-item version of the Chalder Fatigue Scale (CFS). Statistical analyses included multivariable logistic regression for categorical outcomes and analysis of variance for continuous outcomes, adjusting for relevant covariates.

**Results:**

No association was found between VMS consumption and changes in FSS (mean ± standard error 0.05 ± 0.03 vs. -0.06 ± 0.14 for non-consumers and consumers, respectively, *p* = 0.440) and CFS (-0.05 ± 0.06 vs. 0.22 ± 0.28, *p* = 0.388). Similarly, no effect of VMS consumption was found on incidence odds ratio and 95% confidence interval: 1.75 (0.82–3.74), *p* = 0.149 or remission 1.36 (0.49–3.74), *p* = 0.550 of clinical fatigue. Similar findings were obtained for VMDS: FSS 0.06 ± 0.04 vs. 0 ± 0.08, *p* = 0.577; CFS − 0.07 ± 0.08 vs. 0.04 ± 0.15, *p* = 0.545 for non-consumers and consumers, respectively. OR 1.96 (1.20–3.20), *p* = 0.008 and 1.14 (0.57–2.31), *p* = 0.712 for incidence and remission of fatigue. Alternate or persistent VMS/ VMDS consumers had a higher incidence of clinical fatigue and a higher increase in FSS compared with never consumers.

**Conclusion:**

In this population-based sample, we found no consistent association between VMS or VMDS consumption and remission of fatigue. Conversely, VMDS users tended to develop greater fatigue.

**Supplementary Information:**

The online version contains supplementary material available at 10.1007/s00394-025-03615-y.

## Introduction

Fatigue is a common symptom in primary care, usually characterized by low energy, mental exhaustion, and poor muscle endurance. It can have a negative impact on work, family, and society [1]. Fatigue encompasses complex physiological and psychological processes. Biologically, it is closely associated with disorders in energy metabolism. For instance, abnormalities in mitochondrial structure and function can result in inadequate cellular energy production, leading to fatigue [2–4]. Additionally, inflammation, immune system dysregulation, impaired hypothalamic-pituitary-adrenal axis function, and neuroendocrine disorders can negatively impact an individual’s mental state and contribute to fatigue [5–7]. Psychological factors, including chronic stress, depressive symptoms, and sleep disturbances, also play significant roles in the development of fatigue [8–10]. In healthy people, the underlying cause for fatigue is often unknown, and treatment options are scarce; thus, attempts to alleviate or prevent fatigue through nutritional supplements have emerged as a possibility.

The consumption of vitamin/mineral supplements (VMS) and vitamin/mineral and/or dietary supplements (VMDS) is popular among the general population. In Switzerland, 16.8% of the population aged 35 to 75 uses multivitamin and mineral supplements [11]. In the United States, there is a multibillion-dollar dietary supplement industry, at least one-third of which is sold as vitamin/mineral pills and beverages [12]. Research investigating the association between VMS/VMDS and fatigue in the general population is limited and has produced inconsistent findings. Some studies suggest that supplementation with VMS/VMDS can partially alleviate fatigue [13]. However, other research indicates that VMS/VMDS have no significant effect on reducing fatigue [14–16]. Although VMS are beneficial for people with certain clinical diseases [17–20], and with nutritional deficiencies [21, 22], in the general population, most individuals can meet their needs through a healthy diet, so micronutrient supplements are unlikely to be of any benefit [23] and may on the contrary be harmful if taken in excess [12]. Still, most people believe that taking supplements regularly will help them stay healthy, prevent disease, and live longer. This belief is further increased by an aggressive marketing of VMS and VMDS producers, who promote their products as a remedy against fatigue (https://www.naturalia.fr/esprit-bio/parlons-bio/vitamines-contre-la-fatigue, https://nutriandco.com/fr/pages/vitamine-contre-la-fatigue).

Therefore, we aimed to examine the association between VMS, VMDS and fatigue over a mean follow-up of 3.8 years by using data from a population-based prospective study to clarify and solidify recommendations and ensure accurate, useful distribution of information at a population level.

### Participants and methods

### Participants and study design

We used data from CoLaus|PsyCoLaus (www.colauspsycolaus.ch), a population-based study initiated in 2003 with 6733 middle-aged participants from Lausanne, Switzerland to investigate the epidemiology and genetic determinants of cardiovascular risk factors [24]. Recruitment began in 2003 and ended in 2006. The first follow-up was performed between 2009 and 2012, the second between 2014 and 2017 and the third between 2018 and 2021.

Fatigue status was only collected in the second and the third follow-ups. Hence, for this study, data collected from the second (2014–2017) and third (2018–2021) follow-ups were examined, see supplementary Table 1 for more details. In addition, when we analyzed whether participants consumed VMS or VMDS continuously, we included data from the first follow-up of the original study (2009–2012). We define “never” as no consumption at the first (2009–2012) and second (2014–2017) follow-ups of the original study, “alternate” as consumption at the first or second follow-up and “persistent” as consumption at both the first and second follow-ups.

### Vitamin/Mineral/Dietary supplements consumption

Within each survey, participants were asked to report all prescribed and over-the-counter medications and supplements taken during the previous six months. Nonetheless, data on the dosages and frequency of these supplements were not obtained. Vitamin and mineral supplements were defined according to the Swiss compendium (compendium.ch/home/fr, assessed June 2017). If the supplements were not listed in the Swiss compendium, further searches on the internet were conducted. Due to wide differences in the composition of Swiss VMS [23] and to inaccurate reporting (i.e., reporting “multivitamins from producer X” that manufactures six different types of multivitamins), it was not possible to assess the amounts of vitamins and minerals consumed by participants. Dietary supplements were defined as any other supplement that could not be considered as a VMS, such as plant extracts not considered as phytotherapy by the Swiss compendium, cod liver oil, shark cartilage or amino acids.

### Assessment of fatigue

Fatigue levels were assessed once within each survey using the Fatigue Severity Scale (FSS) and the 14-item version of the Chalder Fatigue Scale (CFS).

Fatigue during the previous week was assessed by the 9-item FSS [25]. This questionnaire has been validated for a general healthy population in the Swiss setting [26] and has a high test-retest reliability [27]. The questionnaire is composed of nine questions; responses are graded using a Likert scale from 1 to 7, where 1 indicates strong disagreement and 7 strong agreements. The final score is the mean value of the 9 responses, and a score ≥ 4 is considered as severe fatigue. Fatigue was also assessed using the 14-item CFS [28]. In this study, a binary coding (presence/absence) was applied to the items, at it has been shown that near-maximal scoring of the 14 items constituting the Chalder fatigue scale supports the validity of a two-point scoring rather than the four-point Likert scoring [29].

Changes in fatigue status were computed as follows: first, using dichotomous data (clinical fatigue yes/no) at follow-up 2 (FU2), we assessed the incidence of fatigue at follow-up 3 (FU3) among participants without fatigue at FU2, and remission of fatigue at FU3 among participants with fatigue at FU2, for FSS and CFS. Second, we computed the changes in FSS and CFS as the score at FU3 minus the score at FU2; a positive value denotes an improvement (decrease) in fatigue status. Third, we compared the FSS and CFS scales at FU3 between VMS (VMDS) consumers and non-consumers.

### Relevant covariates

We selected potential confounding factors based on the literature on the relationship between VMS and fatigue. We selected age (years), sex (male/female), education, marital status, weekly alcohol consumption (units), smoking, hypertension (yes/no), diabetes (yes/no), body mass index (BMI) categories and quality of dietary intake, the collection of which is detailed below.

Education was categorized into high (university), middle (high school) and low (apprenticeship + mandatory). Marital status was defined as living alone (single, divorced, widowed) or with a partner. Usual alcohol consumption during the week was self-reported as number of units (glasses of wine, bottles or cans of beer, and shots of spirits) per week. Smoking was self-reported and categorized as never, former (irrespective of the time since quitting smoking) and current.

Body weight and height were measured with participants barefoot and in light indoor clothes. Body weight was measured in kilograms to the nearest 100 g using a Seca^®^ scale (Hamburg, Germany). Height was measured to the nearest 5 mm using a Seca^®^ (Hamburg, Germany) height gauge. BMI was calculated and categorized as normal (< 25 kg/m^2^), overweight ≥ 25 and < 30 kg/m^2^) and obese ≥ 30 kg/m^2^) [30].

Blood pressure (BP) was measured using an Omron^®^ HEM-907 automated oscillometric sphygmomanometer after at least a 10-minute rest in a seated position, and the average of the last two measurements was used. Hypertension was defined by a systolic BP ≥ 140 mm Hg or a diastolic BP ≥ 90 mm Hg or presence of antihypertensive drug treatment [31].

Glucose was assessed by glucose dehydrogenase. Diabetes mellitus (DM) was defined as fasting plasma glucose ≥ 7.0 mmol/L and/or presence of oral hypoglycaemic or insulin treatment [32].

Dietary intake was assessed using a self-administered, semi-quantitative food frequency questionnaire (FFQ) validated on the Geneva population [33]. Briefly, this FFQ assesses the dietary intake of the previous 4 weeks and consists of 97 different food items that account for more than 90% of the intake of calories, proteins, fat, carbohydrates, alcohol, cholesterol, vitamin D and retinol, and 85% of fibre, carotene, and iron. Dietary quality was assessed via the Alternative Healthy Eating Index (AHEI) adapted from McCullough et al. [34]. In our study, the amount of *trans* fat could not be assessed, and we considered all participants taking multivitamins as taking them for a duration ≥ 5 years. Thus, the modified AHEI score ranged between 2.5 and 77.5 instead of 2.5 and 87.5 for the original AHEI score. Higher values represented a healthier diet.

### Exclusion criteria

We excluded participants with missing data for (1) follow-up 3; (2) fatigue; and (3) covariates.

### Statistical analysis

Statistical analyses were conducted using Stata v.18 (Stata Corp, College Station, TX, USA). Descriptive results were expressed as number of participants (percentage) for categorical variables and as average ± standard deviation for continuous variables. Between-group comparisons were conducted using chi-square for categorical variables and student’s t-test for continuous variables. Multivariable analyses were conducted using logistic regression for categorical variables, and results were expressed as odds ratio and (95% confidence interval). Multivariable analyses were conducted using analysis of variance (ANOVA) for quantitative variables and results were expressed as adjusted mean ± standard error. Multivariable analyses were adjusted for the covariates defined previously.

As a significant number of participants were excluded, we conducted sensitivity analyses using inverse probability weighting as defined previously [35]. First, we computed the probability of being included using a logistic model including the variables that differed significantly between included and excluded participants, then we used the inverse of this probability as weight for the multivariable logistic models described previously.

Statistical significance was considered for a two-sided test with *p* < 0.05.

## Results

### Selection of participants

**Fig. 1:**
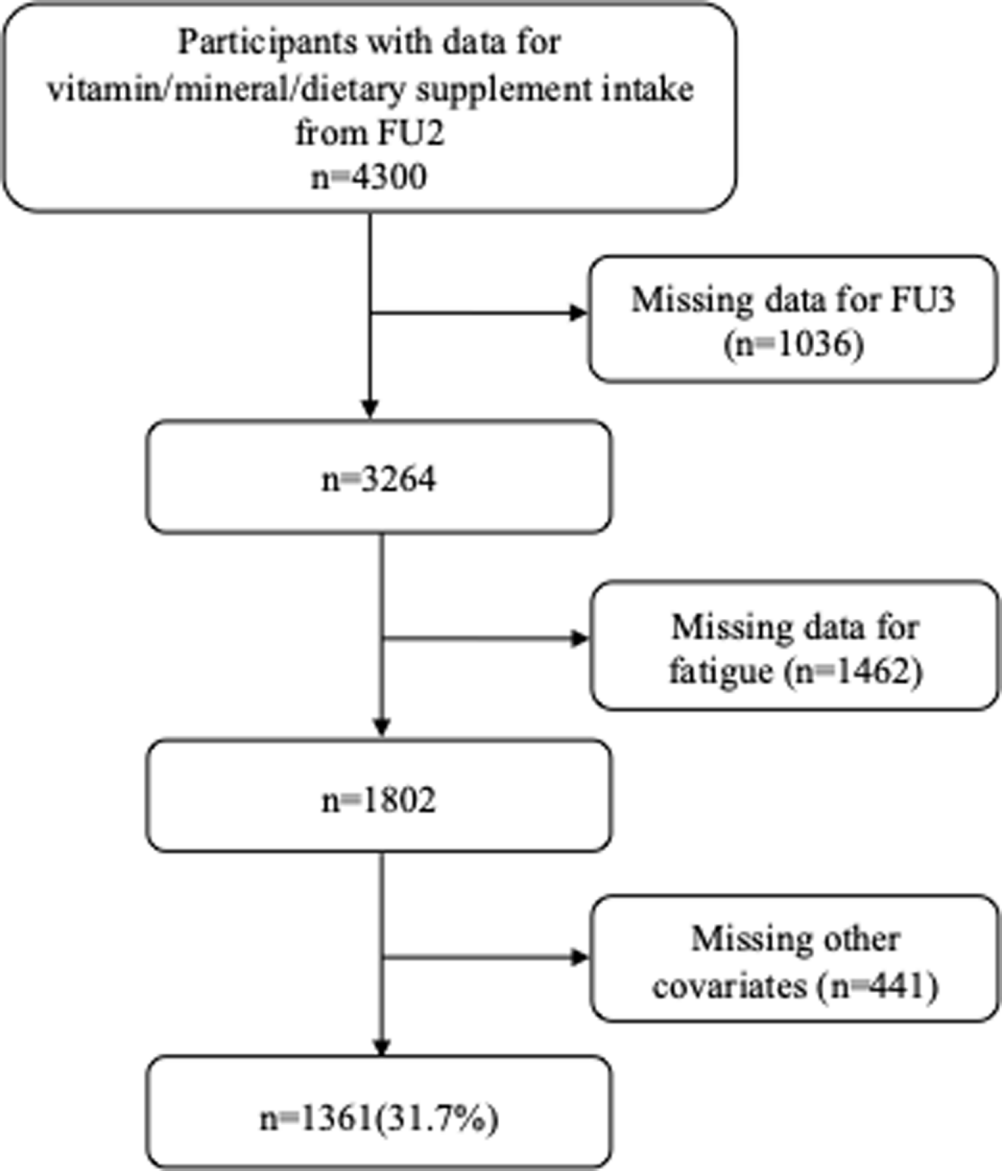
Selection of participants from FU2 (2014–2017). CoLaus study, Lausanne, Switzerland

Of the initial 4300 participants, 1361 (31.7%) were included in the analyses. The reasons for exclusion are indicated in Fig. 1 and the comparison between included and excluded participants is provided in Supplementary Table 2. Compared with the included participants, excluded participants were older, more frequently female, living alone, abstaining from alcohol, and with lower education levels, and more frequently with hypertension, diabetes, obesity, and lower total energy intake. Excluded participants scored higher than included participants regarding FSS and CFS.

Among participants enrolled in the study at the FU2, 79 (5.8%) consumed VMS and 350 (25.7%) consumed VMDS. Table 1 summarizes the characteristics of participants according to VMS and VMDS consumption. Compared with participants who did not consume VMS, participants who consumed VMS were more frequently female, lived alone, and had higher education levels and AHEI (Table 1). Compared with participants who did not consume VMDS, participants who consumed VMDS were older, more frequently female, highly educated, lived alone, abstaining from alcohol, and non-obese, and had a higher AHEI (Table 1).

**Table 1:** Characteristics of the participants, according to VMS use, FU2 (2014–2017) of the CoLaus|PsyColaus study, Lausanne, Switzerland

	VMS non-consumers (*n* = 1282)	VMS consumers (*n* = 79)	*P*-value	VMDS non-consumers (*n* = 1011)	VMDS consumers (*n* = 350)	*P*-value
Age (years)	61.0 ± 9.5	61.0 ± 9.0	0.989	60.7 ± 9.3	62.1 ± 9.7	**0.015**
Women (%)	634 (49.5)	51 (64.6)	**0.009**	458 (45.3)	227 (64.9)	***P*** **< 0.001**
Educational level (%)			**0.028**			**0.028**
Low	572 (44.6)	26 (32.9)		461 (45.6)	137 (39.2)	
Middle	402 (31.4)	24 (30.4)		317 (31.4)	109 (31.1)	
High	308 (24.0)	29 (36.7)		233 (23.0)	104 (29.7)	
Marital status, %			**0.002**			**0.002**
Living alone	406 (31.7)	38 (48.1)		306 (30.3)	138 (39.4)	
Living in a couple	876 (68.3)	41 (51.9)		705 (69.7)	212 (60.6)	
Alcohol consumption, %			0.758			**0.029**
None	238 (18.6)	18 (22.8)		172 (17.0)	84 (24.0)	
1–13 units/week	852 (66.5)	48 (60.8)		681 (67.4)	219 (62.6)	
14–27 units/week	162 (12.6)	11 (13.9)		135 (13.3)	38 (10.8)	
28 + units/week	30 (2.3)	2 (2.5)		23 (2.3)	9 (2.6)	
Smoking categories (%)			0.479			0.371
Never	525 (41.0)	27 (34.2)		421 (41.6)	131 (37.4)	
Former	521 (40.6)	35 (44.3)		404 (40.0)	152 (43.4)	
Current	236 (18.4)	17 (21.5)		186 (18.4)	67 (19.2)	
BMI categories (%)			0.371			**0.027**
Normal	574 (47.8)	32 (40.5)		433 (42.8)	173 (49.4)	
Overweight	518 (40.4)	38 (48.1)		417 (41.3)	139 (39.7)	
Obese	190 (14.8)	9 (11.4)		161 (15.9)	38 (10.9)	
Hypertension (%)	524 (40.9)	30 (38.0)	0.611	416 (41.2)	138 (39.4)	0.573
Diabetes (%)	90 (7.0)	3 (3.8)	0.271	71 (7.0)	22 (6.3)	0.638
Total energy intake (kcal/d)	1735 ± 616	1611 ± 491	0.081	1733 ± 613	1713 ± 600	0.595
AHEI	31.9 ± 10.0	35.7 ± 10.1	**0.001**	31.2 ± 9.7	34.7 ± 10.6	***P*** **< 0.001**

Results are expressed as number of participants (column percentage) for categorical variables and as average ± standard deviation for continuous variables. Between-group comparisons performed using chi-square for categorical variables and student’s t-test for continuous variables. Abbreviations: VMS: Vitamin/mineral supplements; VMDS: Vitamin/mineral and/or dietary supplements; BMI: Body mass index; AHEI: Alternate Healthy Eating Index

### Association between VMS and VMDS consumption and change in fatigue indicators

**Fig. 2:**
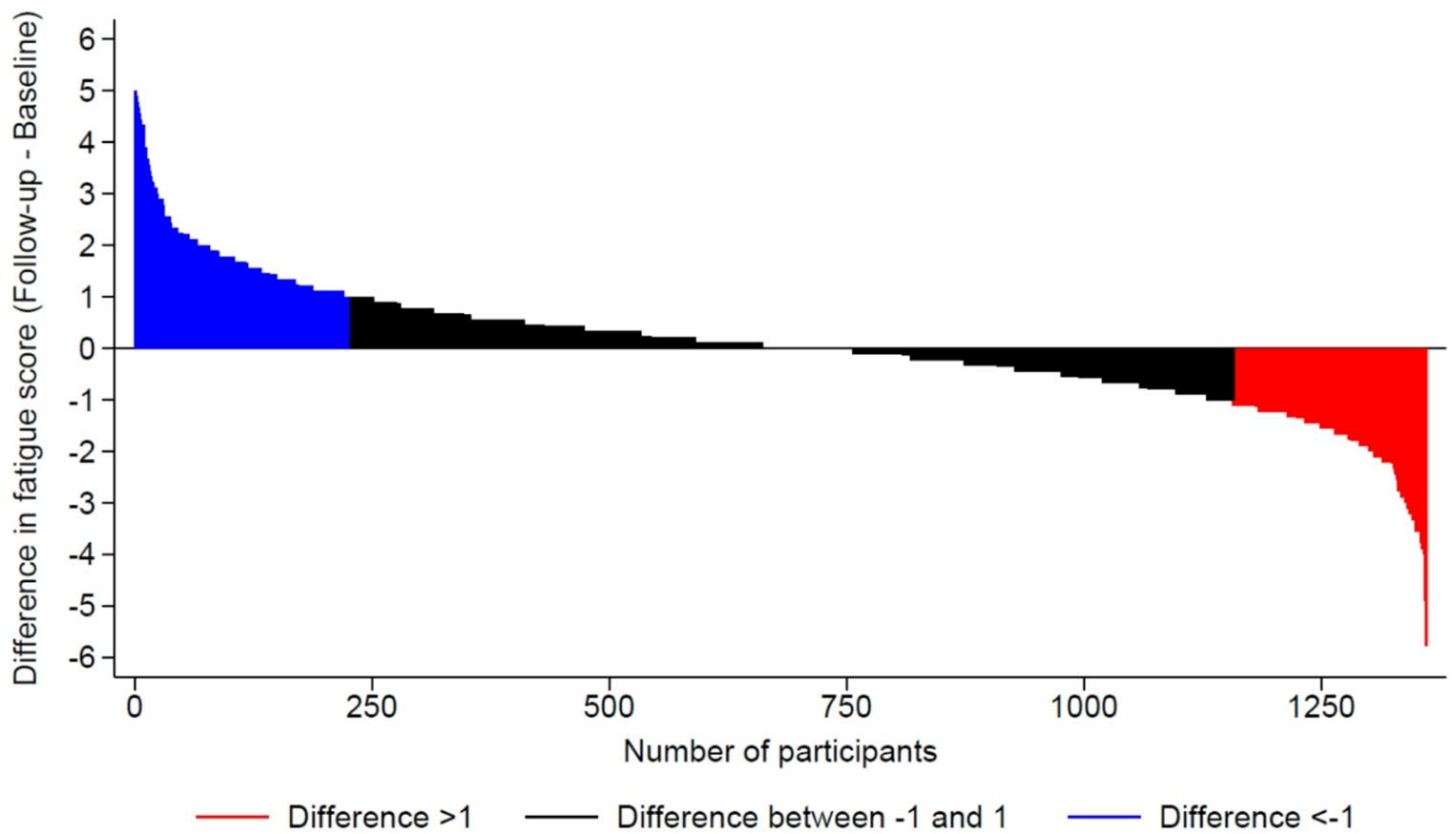
Differences in Fatigue Severity Scale between the FU3 and the FU2

**Fig. 3:**
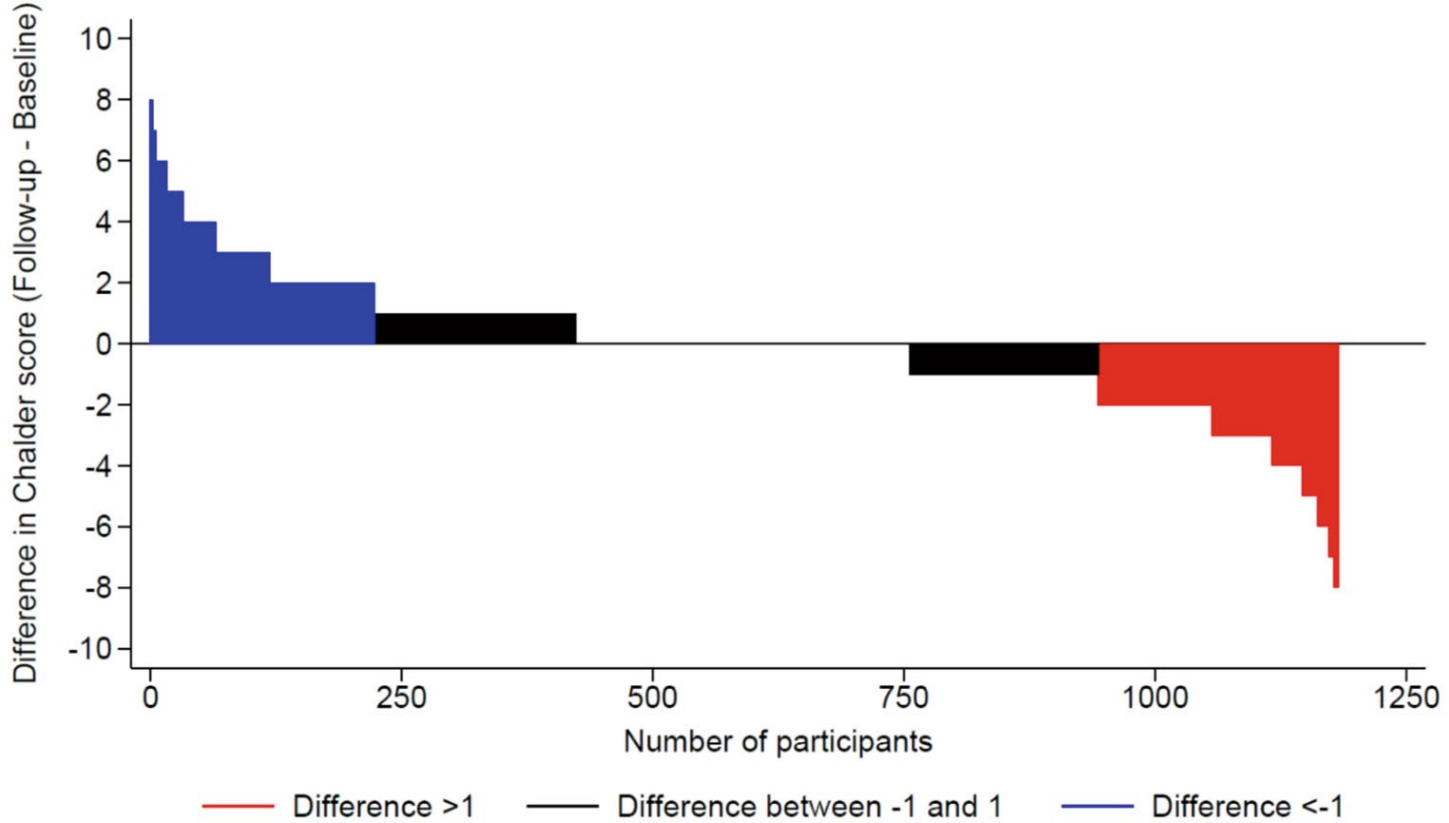
Differences in Chalder Fatigue Scale between the FU3 and the FU2

A summary of the association between VMS consumption and changes in fatigue indicators after a median follow-up of 3.8 (interquartile range: 3.6–3.9) years is shown in Table 2. In bivariate and multivariable analyses, there was no association between VMS consumption and changes of FSS and CFS, incidence or remission of clinical fatigue, and fatigue scores at FU3 (Table 2). Figures 2 and 3 show the differences between FU3 and FU2 in FSS and CFS, respectively.

A summary of the association between VMDS consumption and changes in fatigue indicators is shown in Table 3. In bivariate and multivariable analyses, there was no association between VMDS consumption and changes of CFS and FSS, remission of clinical fatigue (Table 3). However, participants consuming VMDS had higher incidence of clinical fatigue and CFS (Table 3).

Using inverse probability weighting to correct for exclusions led to similar findings (supplementary Table 3).

**Table 2:** Bivariate and multivariable associations between VMS consumption and changes in fatigue, CoLaus|PsyColaus study, Lausanne, Switzerland

	Bivariate			Multivariable		
	VMS non-consumers	VMS consumers	*P*-value	VMS non-consumers	VMS consumers	*P*-value
Incident clinical fatigue^1^	1 (ref.)	1.63 (0.78–3.42)	0.193	1 (ref.)	1.75 (0.82–3.74)	0.149
Remission of clinical fatigue ^2^	1 (ref.)	1.17 (0.47–2.92)	0.742	1 (ref.)	1.36 (0.49–3.74)	0.550
Changes in Fatigue score^3^	0.05 ± 0.03	-0.05 ± 0.14	0.462	0.05 ± 0.03	-0.06 ± 0.14	0.440
Changes in Chalder score^4^	-0.05 ± 0.06	0.18 ± 0.28	0.415	-0.05 ± 0.06	0.22 ± 0.28	0.338
Fatigue score^5^	2.83 ± 1.38	2.99 ± 1.41	0.314	2.83 ± 0.04	2.95 ± 0.16	0.439
Chalder score^5^	5.54 ± 2.47	6.13 ± 2.79	0.069	5.54 ± 0.07	6.02 ± 0.31	0.139

Results are expressed as average ± standard deviation for bivariate analysis and as multivariable-adjusted average ± standard error or odds ratio and 95% confidence interval. Between group comparison using ANOVA or logistic regression, multivariable analysis adjusted for age, sex, BMI categories (normal, overweight, obese), education(low/medium/high), marital status (alone, in couple), smoking (never, former, current), alcohol consumption (none, 1–13, 14–27 and 28 + per week), hypertension (yes, no), diabetes (yes, no), total energy intake (continuous), AHEI (continuous). Abbreviations: VMS: Vitamin/mineral supplements; 1: among participants who did not have clinical fatigue at FU2. 2: among participants with clinical fatigue at FU2. 3: The change in Fatigue score is the Fatigue score at FU3 minus the fatigue score at FU2. 4: The change in Chalder score is the Chalder score at FU3 minus the fatigue score at FU2. 5: The value of the FU3

**Table 3:** Bivariate and multivariable associations between VMDS consumption and changes in fatigue, CoLaus|PsyColaus study, Lausanne, Switzerland

	Bivariate			Multivariable		
	VMDS non-consumers	VMDS consumers	*P*-value	VMDS non-consumers	VMDS consumers	*P*-value
Incident clinical fatigue^1^	1 (ref.)	1.64 (1.07–2.49)	**0.022**	1 (ref.)	1.96 (1.20–3.20)	**0.008**
Remission of clinical fatigue^2^	1 (ref.)	0.85 (0.50–1.44)	0.542	1 (ref.)	1.14 (0.57–2.31)	0.712
Changes in Fatigue score^3^	0.06 ± 0.04	0.01 ± 0.06	0.562	0.06 ± 0.04	0 ± 0.08	0.577
Changes in Chalder score^4^	0 ± 0.07	-0.16 ± 0.13	0.269	-0.07 ± 0.08	0.04 ± 0.15	0.545
Fatigue score^5^	2.77 ± 1.35	3.01 ± 1.48	**0.005**	2.78 ± 0.05	2.99 ± 0.09	0.053
Chalder score^5^	5.42 ± 2.41	6.03 ± 2.67	***P*** **< 0.001**	5.44 ± 0.09	5.98 ± 0.17	**0.008**

Results are expressed as average ± standard deviation or number of participants (column percentage) for bivariate analysis and as multivariable-adjusted average ± standard error or odds ratio and 95% confidence interval. Between group comparison using ANOVA or logistic regression, multivariable analysis adjusted for age, sex, BMI categories (normal, overweight, obese), education(low/medium/high), marital status (alone, in couple), smoking (never, former, current), alcohol consumption (none, 1–13, 14–27 and 28 + per week), hypertension (yes, no), diabetes (yes, no), total energy intake (continuous), AHEI (continuous). Abbreviations: VMDS: Vitamin/mineral and/or dietary supplements; 1: among participants who did not have clinical fatigue at FU2. 2: among participants with clinical fatigue at FU2. 3: The change in Fatigue score is the Fatigue score at FU3 minus the fatigue score at FU2. 4: The change in Chalder score is the Chalder score at FU3 minus the fatigue score at FU2. 5: The value of the FU3

### Association between VMS and VMDS (non) persistent consumption and change in fatigue indicators

The association between never, alternate, or persistent VMS and VMDS consumption and fatigue indicators at follow-up are indicated in Table 4. For VMS, multivariable analysis showed that alternate or persistent VMS consumers had a higher incidence of clinical fatigue compared with never consumers. Similar findings were obtained for VMDS, alternate or persistent consumers having a higher incidence of clinical fatigue than never consumers and FSS. Using inverse probability weighting to correct for exclusions led to similar findings (supplementary Table 4).

**Table 4:** The association between VMS and VMDS consumption based on the FU1 (2009–2012) and the FU2 (2014–2017) of the original study, and evolution of fatigue between FU2 (2014–2017) and FU3 (2018–2021), CoLaus|PsyColaus study, Lausanne, Switzerland

	Never	Alternate	Persistent	*P*-value for trend
For VMS consumption				
Incident clinical fatigue^1^	1 (ref.)	1.27 (0.58–2.79)	3.81 (1.31–11.12)	**0.014**
Remission of clinical fatigue ^2^	1 (ref.)	1.56 (0.66–3.68)	nc	nc
Changes in Fatigue score^3^	0.05 ± 0.03	-0.12 ± 0.12	0.57 ± 0.27	0.053
Changes in Chalder score^4^	-0.06 ± 0.07	0.07 ± 0.24	0.72 ± 0.53	0.145
Fatigue score^5^	2.81 ± 0.04	3.05 ± 0.14	3.13 ± 0.3	0.291
Chalder score^5^	5.53 ± 0.07	6.02 ± 0.27	5.71 ± 0.59	0.759
**For VMDS consumption**				
Incident clinical fatigue^1^	1 (ref.)	1.36 (0.82–2.26)	2.73 (1.42–5.27)	**0.003**
Remission of clinical fatigue ^2^	1 (ref.)	1.22 (0.62–2.4)	0.7 (0.22–2.17)	0.531
Changes in Fatigue score^3^	0.06 ± 0.04	-0.03 ± 0.07	0.14 ± 0.12	0.544
Changes in Chalder score^4^	-0.07 ± 0.08	-0.02 ± 0.14	0.14 ± 0.24	0.427
Fatigue score^5^	2.79 ± 0.05	2.86 ± 0.08	3.15 ± 0.14	**0.017**
Chalder score^5^	5.45 ± 0.09	5.83 ± 0.16	5.9 ± 0.27	0.119

Nc, not computable. Results are expressed as multivariable-adjusted average ± standard error or odds ratio and 95% confidence interval. Between group comparison using ANOVA or logistic regression adjusting for age, sex, BMI categories (normal, overweight, obese), education(low/medium/high), marital status (alone, in couple), smoking (never, former, current), alcohol consumption (none, 1–13, 14–27 and 28 + per week), hypertension (yes, no), diabetes (yes, no), total energy intake (continuous), alternate healthy eating index (continuous). Abbreviations: VMS: Vitamin/mineral supplements; VMDS: Vitamin/mineral and/or dietary supplements. 1: among participants who did not have clinical fatigue at FU2. 2: among participants with clinical fatigue at FU2. 3: The change in Fatigue score is the Fatigue score at FU3 minus the fatigue score at FU2. 4: The change in Chalder score is the Chalder score at FU3 minus the fatigue score at FU2. 5: The value of the FU3

## Discussion

In this study, we investigated the association between VMS consumption and VMDS and fatigue in community-dwelling Swiss adults. Our prospective study failed to find any evidence of a beneficial effect of VMS and VMDS consumption and changes in fatigue status.

### Association between VMS and VMDS and fatigue

There are few studies on the potential association between vitamins and minerals and dietary supplements and fatigue in the general population, and the conclusions are mixed. Therefore, in Supplementary Table 5 we summarize the effects of oral vitamins and minerals and dietary supplements on fatigue, according to literature.

#### Vitamin and mineral supplements

Several studies have shown that individual supplements of vitamin B1 [36], vitamin B12 [37], vitamin C [17, 38], vitamin D [21, 39, 40], vitamin E [41], as well as iron [42, 43], and zinc [44] can relieve fatigue symptoms. Similarly, supplementing with a variety of vitamins and minerals can also reduce fatigue to a certain extent [13].

However, contrary to the above positive results, several studies found no effect of vitamin or mineral supplements in reducing fatigue. A randomized controlled trial showed that oral vitamin D3 did not improve fatigue in patients with chronic fatigue syndrome [14]. Data from Brouwers et al. found no statistically significant relationship between multivitamins and mineral supplementation and symptoms of chronic fatigue syndrome [15]. Also, a meta-analysis revealed that vitamin and mineral status did not lead to clinical improvement in chronic fatigue syndrome [16].

We found that, for participants devoid of fatigue at FU2, after 3.8 years of follow-up, VMS consumption had no effect on the incidence of fatigue. Moreover, for participants who presented with fatigue at FU2, after 3.8 years of follow-up, consuming VMS had no effect on remission. Hence, our results confirm the negative findings of previous studies [14–16].

However, interestingly, when we categorized VMS consumption as never, alternate, and persistent, and among participants devoid of fatigue at FU2, participants who consumed VMS alternatively or persistently had a higher prevalence of clinical fatigue at follow-up and a higher increase in FSS score than participants who never consumed VMS. One possible explanation for this finding is that some participants consume VMS to avoid fatigue in the future. Still, as no information was collected regarding the reason to consume VMS, further studies are needed to clarify this issue.

#### Dietary supplements

As shown in Supplementary Table 5, supplements such as dietary polysaccharides [45], coenzyme Q10 [46, 47], omega-3 polyunsaturated fatty acids [48], dietary nitrate [49, 50], soluble dietary fiber [51], astaxanthin and sesamin [52], acetyl L-carnitine [53], superoxide dismutase-melon concentrate supplementation [54], and gut probiotics [55] have been suggested to reduce fatigue burden. However, two systematic reviews found limited evidence that dietary supplements can help relieve symptoms of chronic fatigue syndrome, findings being inconsistent across studies [56, 57]. Another study showed no significant association between dietary supplement intake and fatigue severity or functional impairment [58].

In our study, among participants devoid of fatigue at FU2, those who consumed VMDS had a higher incidence of fatigue and had higher FSS and CFS scores after 3.8 years of follow-up than those who did not consume VMDS. Similar findings were obtained when we categorized VMDS consumption as never, alternate, and persistent, persistent consumers having a higher incidence of fatigue and higher FSS scores after 3.8 years of follow-up.

For participants who presented with fatigue at FU2, after 3.8 years of follow-up, consuming VMDS had no effect on remission, and similar findings were obtained when we categorized VMDS consumption as never, alternate, and persistent. Again, a possible explanation is that participants consume VMDS in the hope of reducing the fatigue that may occur.

### Potential mechanisms and explanations

The reasons why this study failed to find a beneficial effect of VMS and VMDS on fatigue may include the following: (1) Most of the general population already meets their micronutrient requirements through a balanced diet. Therefore, additional supplement intake may not bring additional benefits in terms of fatigue reduction. In some cases, excessive intake of certain vitamins and minerals may even lead to adverse reactions, which may aggravate fatigue symptoms [12]. (2) The association we observed between continuous or occasional use of supplements and increased fatigue prevalence may reflect reverse causation. That is, individuals who feel tired are more likely to use supplements in the hope of relieving symptoms, rather than supplements causing fatigue. This self-selection bias complicates the interpretation of observational data. (3) Fatigue is a multifactorial condition that is affected by a combination of biological, psychological, and social factors. VMS and VMDS may not address the complex mechanisms that cause fatigue, such as chronic inflammation, hormone imbalance, or psychological stress. Effective fatigue management may require comprehensive lifestyle adjustments, psychological interventions, and targeted medical treatments. (4) The effectiveness of supplements may vary depending on their formulation, dosage, and bioavailability. Differences in product quality and the presence of other active or inactive ingredients may affect their potential effects on fatigue, leading to inconsistent study results.

### Strengths and limitations

This is a prospective study with a large population-based sample, making our findings more generalizable than studies conducted in experimental settings or within specific populations such as athletes [36, 51]. Second, we used two methods to quantify/classify fatigue, leading to similar findings.

Our study also has some limitations. First, the inaccurate reporting of VMS composition and dosages by the participants prevented us from assessing the amount of vitamins and minerals consumed by participants. Second, the study was conducted in a limited geographical location, which might not be representative of the entire country. Still, the prevalence of VMS or VMDS users was similar to other countries [59, 60]. Third, possible reverse causation (i.e., participants taking VMS/VMDS to prevent fatigue) cannot be excluded, although it is unlikely that participants consuming VMS/VMDS did so to prevent fatigue over a 5-year period.

## Conclusion

In this Swiss population-based sample, no consistent association was found between the consumption of VMS and VMDS and fatigue incidence or remission. Therefore, in the general population, consumption of VMS and VMDS does not have a significant impact on fatigue.

## Electronic supplementary material

Below is the link to the electronic supplementary material.

Supplementary Material 1

Supplementary Material 2

## Data Availability

The data of CoLaus|PsyCoLaus study used in this article cannot be fully shared as they contain potentially sensitive personal information on participants. According to the Ethics Committee for Research of the Canton of Vaud, sharing these data would be a violation of the Swiss legislation with respect to privacy protection. However, coded individual-level data that do not allow researchers to identify participants are available upon request to researchers who meet the criteria for data sharing of the CoLaus|PsyCoLaus Datacenter (CHUV, Lausanne, Switzerland). Any researcher affiliated to a public or private research institution who complies with the CoLaus|PsyCoLaus standards can submit a research application to research.colaus@chuv.ch or research.psycolaus@chuv.ch. Proposals requiring baseline data only, will be evaluated by the baseline (local) Scientific Committee (SC) of the CoLaus and PsyCoLaus studies. Proposals requiring follow-up data will be evaluated by the follow-up (multicentric) SC of the CoLaus|PsyCoLaus cohort study. Detailed instructions for gaining access to the CoLaus|PsyCoLaus data used in this study are available at www.colaus-psycolaus.ch/professionals/how-to-collaborate.
