# Supplementary material for: Vitamin and mineral supplements and fatigue: a prospective study
Source: Eur J Nutr. 2025 Feb 22;64(2):98. doi: 10.1007/s00394-025-03615-y (PMC11889016; doi:10.1007/s00394-025-03615-y)
Supplement: Supplementary file 1 — Supplementary Material 1 [file 394_2025_3615_MOESM1_ESM.docx]

**Supplementary information**

**Supplementary Table 1:** Data used in the study, CoLaus|PsyColaus study, Lausanne, Switzerland.

| **Colaus original study** | | **Fatigue Data** | **Vitamin/Mineral/Diet Supplement Data** |
| --- | --- | --- | --- |
| Baseline | 2003-2006 |  |  |
| Follow-up 1 | 2009-2012 |  | X |
| Follow-up 2 | 2014-2017 | X | X |
| Follow-up 3 | 2018-2021 | X | X |

**Supplementary Table 2:** Characteristics of included and excluded participants, FU2 (2014-2017), CoLaus|PsyColaus study, Lausanne, Switzerland.

|  | **Included**  (n=1361) | **Excluded**  (n=2939) | ***P*-value** |
| --- | --- | --- | --- |
| Age (years) | 61.0 ± 9.4 | 63.5 ± 10.7 | **<0.001** |
| Women (%) | 685 (50.3) | 1674 (57.0) | **<0.001** |
| Educational level (%) |  |  | **<0.001** |
| Low | 598 (43.9) | 1612 (54.9) |  |
| Middle | 426 (31.3) | 715 (24.4) |  |
| High | 337 (24.8) | 609 (20.7) |  |
| Marital status, % |  |  | **0.003** |
| Living alone | 444 (32.6) | 821 (37.5) |  |
| Living in a couple | 917 (67.4) | 1366 (62.5) |  |
| Alcohol consumption, % |  |  | **<0.001** |
| None | 256 (18.8) | 750 (30.6) |  |
| 1-13/week | 900 (66.1) | 1382 (56.3) |  |
| 14-27/week | 173 (12.7) | 258 (10.5) |  |
| 28+/week | 32 (2.4) | 63 (2.6) |  |
| Smoking categories (%) |  |  | 0.460 |
| Never | 552 (40.6) | 1121 (42.4) |  |
| Former | 556 (40.8) | 1030 (38.9) |  |
| Current | 253 (18.6) | 495 (18.7) |  |
| BMI categories (%) |  |  | **<0.001** |
| Normal | 606 (44.5) | 1109 (40.4) |  |
| Overweight | 556 (40.9) | 1075 (39.1) |  |
| Obese | 199 (14.6) | 564 (20.5) |  |
| Hypertension (%) | 554 (40.7) | 1413 (49.6) | **<0.001** |
| Diabetes (%) | 93 (6.8) | 343 (12.3) | **<0.001** |
| Total energy intake (kcal/d) | 1728 ± 610 | 1630 ± 681 | **<0.001** |
| Fatigue_score | 2.8 ± 1.4 | 3.0 ± 1.5 | **0.002** |
| Chalder_score | 5.6 ± 2.5 | 6.0 ± 2.6 | **<0.001** |
| Clinical fatigue (%) | 265 (19.5) | 397 (24.1) | **0.002** |
| AHEI | 32.1 ± 10.0 | 32.2 ± 10.0 | 0.877 |

Results are expressed as number of participants (column percentage) for categorical variables and as average ± standard deviation for continuous variables. Between-group comparisons performed using chi-square for categorical variables and student’s t-test for continuous variables. Abbreviations: BMI: Body mass index; AHEI: Alternate Healthy Eating Index.

**Supplementary Table 3**: multivariable associations between VMS and VMDS consumption and changes in fatigue, CoLaus|PsyColaus study, Lausanne, Switzerland, using inverse probability weighting.

|  | **Non-consumers** | **Consumers** | ***P* -value** |
| --- | --- | --- | --- |
| **For VMS consumption** |  |  |  |
| Incident clinical fatigue^1^ | 1 (ref.) | 1.63 (0.70 - 3.76) | 0.255 |
| Remission of clinical fatigue ^2^ | 1 (ref.) | 1.37 (0.49 - 3.86) | 0.551 |
| **For VMDS consumption** |  |  |  |
| Incident clinical fatigue^1^ | 1 (ref.) | 1.90 (1.14 - 3.16) | **0.014** |
| Remission of clinical fatigue ^2^ | 1 (ref.) | 1.18 (0.58 - 2.40) | 0.654 |

Results are expressed as multivariable-adjusted odds ratio and 95% confidence interval. Between group comparison using logistic regression adjusted for age, sex, BMI categories (normal, overweight, obese), education(low/medium/high), marital status (alone, in couple), smoking (never, former, current), alcohol consumption (none, 1-13, 14-27 and 28+ per week), hypertension (yes, no), diabetes (yes, no), total energy intake (continuous), AHEI (continuous). Abbreviations: VMS: Vitamin/mineral supplements; 1: among participants who did not have clinical fatigue at FU2. 2: among participants with clinical fatigue at FU2.

**Supplementary Table 4**: The association between VMS and VMDS consumption based on the first (2009-2012) and the second (2014-2017) follow-ups, and evolution of fatigue, CoLaus|PsyColaus study, Lausanne, Switzerland, using inverse probability weighting.

|  | **Never** | **Alternate** | **Persistent** | ***P* -value for trend** |
| --- | --- | --- | --- | --- |
| **For VMS consumption** |  |  |  |  |
| Incident clinical fatigue^1^ | 1 (ref.) | 1.51 (0.63 - 3.59) | 3.33 (0.93 - 11.8) | 0.064 |
| Remission of clinical fatigue ^2^ | 1 (ref.) | 1.39 (0.54 - 3.56) | nc | nc |
| **For VMDS consumption** |  |  |  |  |
| Incident clinical fatigue^1^ | 1 (ref.) | 1.68 (0.97 - 2.92) | 2.49 (1.21 - 5.15) | **0.014** |
| Remission of clinical fatigue ^2^ | 1 (ref.) | 1.16 (0.55 - 2.46) | 0.47 (0.11 - 2.04) | 0.314 |

Nc, not computable. Results are expressed as multivariable-adjusted odds ratio and 95% confidence interval. Between group comparison logistic regression adjusting for age, sex, BMI categories (normal, overweight, obese), education(low/medium/high), marital status (alone, in couple), smoking (never, former, current), alcohol consumption (none, 1-13, 14-27 and 28+ per week), hypertension (yes, no), diabetes (yes, no), total energy intake (continuous), alternate healthy eating index (continuous). Abbreviations: VMS: Vitamin/mineral supplements; VMDS: Vitamin/mineral and/or dietary supplements. 1: among participants who did not have clinical fatigue at FU2. 2: among participants with clinical fatigue at FU2.

**Supplementary Table 5: Studies about vitamin/mineral and/or dietary supplements and fatigue.**

| Reference | Study Design | Group | Treatment | No. of Participants | Main Findings |
| --- | --- | --- | --- | --- | --- |
| Suzuki et al., 1996[1] | Non-randomized study | Male athletes | Vitamin B1 | 16 | Subjective fatigue is significantly reduced after exercise |
| Van Campen et al., 2019[2] | Clinical research | Chronic fatigue syndrome patients | Vitamin B12 | 51 | After treatment, participants’ fatigue scale improved |
| Suh et al., 2012[3] | Double-blind randomized controlled trial | healthy volunteers | Vitamin C | 141 | High dose intravenous vitamin C proved to be effective against fatigue in this study |
| Huck et al., 2013[4] | Placebo-controlled pilot trial | Obese adults | Vitamin C | 20 | The general fatigue score was significantly decreased in the vitamin C group compared to the control group (p = 0.001) |
| Al-Eisa et al., 2016[5] | Cross-sectional study | Healthy older subjects | Vitamin D | 85 | Vitamin D may prevent muscle fatigue |
| Roy et al., 2014[6] | Prospective non-randomized therapeutic study | Adult patients with fatigue | Vitamin D | 174 | Correction of Low Vitamin D Improves Fatigue (P < 0.001) |
| Nowak et al.,2016[7] | Double-blind placebo-controlled clinical trial | Healthy persons with vitamin D deficiency | Vitamin D | 120 | Vitamin D treatment significantly improved fatigue in otherwise healthy persons with vitamin D deficiency. |
| Witham et al., 2015[8] | Parallel-group, double-blind, randomized placebo-controlled trial | Patients with chronic fatigue syndrome | Vitamin D | 50 | High-dose oral vitamin D3 did not improve fatigue in chronic fatigue syndrome. |
| Takemoto et al., 2015[9] | Placebo-controlled, double-blind, parallel-group study | Healthy adults | Sesame Lignans and Vitamin E | 309 | The fatigue status of the experimental group was significantly improved (p<0.01) |
| Patterson et al., 2001[10] | Randomized controlled trial | Iron deficient women | Iron | 44 | Treatment of iron deficiency with either supplementation or a high iron diet results in decreased fatigue |
| Favrat et al., 2014[11] | Randomized, placebo-controlled, single-blinded study | Iron deficient women | Iron | 290 | A single infusion of ferric carboxymaltose improved fatigue, in iron-deficient women with normal or borderline hemoglobin |
| Afzali et al., 2021[12] | A parallel clinical trial | Healthy older subjects | Zinc | 150 | Zinc supplementation significantly reduced fatigue (mean difference: -10.41 vs. 1.37, P<0.001) |
| Maric et al., 2014[13] | Prospective study | Women of reproductive age | Multivitamin mineral supplementation | 38 | Fatigue significantly improved (p=0.0009) |
| Brouwers et al., 2002[14] | Double-blind randomized controlled trial | Patients with chronic fatigue syndrome | Dietary supplements | 53 | No significant differences were found between the placebo and the treated group |
| Joustra et al.,2017[15] | A systematic review and meta-analysis | Chronic fatigue syndrome and fibromyalgia syndrome | Vitamins and minerals | 5 RCTs and 40 observational studies | Little evidence was found to support the hypothesis that vitamin and mineral deficiencies play a role |
| Castro-Marrero et al.,  2021[16] | Prospective, randomized, double-blind, placebo-controlled trial | Patients with chronic fatigue syndrome | Coenzyme Q10 and NADH | 207 | A significant reduction in cognitive fatigue perception (p<0.001) from baseline were observed within the experimental group |
| Mizuno et al., 2008[17] | Double-blinded, placebo-controlled, three crossover design | Healthy volunteers | Coenzyme Q10 | 17 | Significant alleviation of subjective fatigue sensation measured on a visual analogue scale in the 300mg coenzyme Q10–administered group after |
| Maes et al., 2005[18] | Cross-sectional study | Patients with chronic fatigue syndrome (CFS) and heathy participants | Omega3 | 34 | Total omega3/omega6 ratios were significantly lower in CFS patients than in normal controls. |
| Husmann et al.,2019[19] | Time-matched trial | Active males | Dietary Nitrate  (beetroot juice) | 12 | Dietary Nitrate Supplementation reducing muscle Fatigue |
| Zoughaib et al.,2023[39] | Double-blind, placebo-controlled, crossover design | Older women | Dietary Nitrate  (beetroot juice) | 18 | No effect on either the fatigability or recoverability of muscle in this population |
| Hadžić et al., 2023[21] | Double-blind, placebo-controlled trial | Healthy male basketball players | Soluble dietary fibre | 18 | Fibre supplementation could have a significant effect on reducing the rating of perceived exertion |
| Imai et al., 2018[22] | Double blind placebo controlled two-way crossover study | Healthy volunteers | Astaxanthin and sesamin | 24 | Astaxanthin and sesamin supplementation was associated with significantly improved recovery from mental fatigue |
| Carillon et al., 2014[23] | Randomized, double-blind, placebo-controlled trial | Healthy volunteers | Superoxide dismutase (SOD)-melon concentrate supplementation | 61 | SOD-melon concentrate significantly decreased physical and mental fatigue |
| Malaguarnera et al.,2008[24] | Single center, randomized, double blind, comparative clinical trial | Elderly patients with fatigue | Acetyl L-carnitine (ALC) | 96 | Administering ALC may reduce both physical and mental fatigue in elderly and improves both the cognitive status and physical functions. |
| Sullivan et al., 2009[25] | Open pilot study | Patients with chronic fatigue syndrome | Probiotic | 15 | 6 out of 15 patients reported that they had improved according to the assessment described |

**References**

1. Suzuki M, Itokawa Y (1996) Effects of thiamine supplementation on exercise-induced fatigue. Metab Brain Dis 11:95–106. https://doi.org/10.1007/bf02080935

2. van Campen CLM, Riepma K, Visser FC (2019) Open Trial of Vitamin B12 Nasal Drops in Adults With Myalgic Encephalomyelitis/Chronic Fatigue Syndrome: Comparison of Responders and Non-Responders. Front Pharmacol 10:1102. https://doi.org/10.3389/fphar.2019.01102

3. Suh SY, Bae WK, Ahn HY, et al (2012) Intravenous vitamin C administration reduces fatigue in office workers: a double-blind randomized controlled trial. Nutr J 11:7. https://doi.org/10.1186/1475-2891-11-7

4. Huck CJ, Johnston CS, Beezhold BL, Swan PD (2013) Vitamin C status and perception of effort during exercise in obese adults adhering to a calorie-reduced diet. Nutrition 29:42–45. https://doi.org/10.1016/j.nut.2012.01.021

5. Al-Eisa ES, Alghadir AH, Gabr SA (2016) Correlation between vitamin D levels and muscle fatigue risk factors based on physical activity in healthy older adults. Clin Interv Aging 11:513–522. https://doi.org/10.2147/cia.S102892

6. Roy S, Sherman A, Monari-Sparks MJ, et al (2014) Correction of Low Vitamin D Improves Fatigue: Effect of Correction of Low Vitamin D in Fatigue Study (EViDiF Study). N Am J Med Sci 6:396–402. https://doi.org/10.4103/1947-2714.139291

7. Nowak A, Boesch L, Andres E, et al (2016) Effect of vitamin D3 on self-perceived fatigue: A double-blind randomized placebo-controlled trial. Medicine (Baltimore) 95:e5353. https://doi.org/10.1097/md.0000000000005353

8. Witham MD, Adams F, McSwiggan S, et al (2015) Effect of intermittent vitamin D3 on vascular function and symptoms in chronic fatigue syndrome--a randomised controlled trial. Nutr Metab Cardiovasc Dis 25:287–294. https://doi.org/10.1016/j.numecd.2014.10.007

9. Takemoto D, Yasutake Y, Tomimori N, et al (2015) Sesame Lignans and Vitamin E Supplementation Improve Subjective Statuses and Anti-Oxidative Capacity in Healthy Humans With Feelings of Daily Fatigue. Glob J Health Sci 7:1–10. https://doi.org/10.5539/gjhs.v7n6p1

10. Patterson AJ, Brown WJ, Roberts DC (2001) Dietary and supplement treatment of iron deficiency results in improvements in general health and fatigue in Australian women of childbearing age. J Am Coll Nutr 20:337–342. https://doi.org/10.1080/07315724.2001.10719054

11. Favrat B, Balck K, Breymann C, et al (2014) Evaluation of a single dose of ferric carboxymaltose in fatigued, iron-deficient women--PREFER a randomized, placebo-controlled study. PLoS One 9:e94217. https://doi.org/10.1371/journal.pone.0094217

12. Afzali A, Goli S, Moravveji A, et al (2021) The effect of zinc supplementation on fatigue among elderly community dwellers: A parallel clinical trial. Health Sci Rep 4:e301. https://doi.org/10.1002/hsr2.301

13. Maric D, Brkic S, Tomic S, et al (2014) Multivitamin mineral supplementation in patients with chronic fatigue syndrome. Med Sci Monit 20:47–53. https://doi.org/10.12659/msm.889333

14. Brouwers FM, Van Der Werf S, Bleijenberg G, et al (2002) The effect of a polynutrient supplement on fatigue and physical activity of patients with chronic fatigue syndrome: a double-blind randomized controlled trial. Qjm 95:677–683. https://doi.org/10.1093/qjmed/95.10.677

15. Joustra ML, Minovic I, Janssens KAM, et al (2017) Vitamin and mineral status in chronic fatigue syndrome and fibromyalgia syndrome: A systematic review and meta-analysis. PLoS One 12:e0176631. https://doi.org/10.1371/journal.pone.0176631

16. Castro-Marrero J, Segundo MJ, Lacasa M, et al (2021) Effect of Dietary Coenzyme Q10 Plus NADH Supplementation on Fatigue Perception and Health-Related Quality of Life in Individuals with Myalgic Encephalomyelitis/Chronic Fatigue Syndrome: A Prospective, Randomized, Double-Blind, Placebo-Controlled Trial. Nutrients 13:. https://doi.org/10.3390/nu13082658

17. Mizuno K, Tanaka M, Nozaki S, et al (2008) Antifatigue effects of coenzyme Q10 during physical fatigue. Nutrition 24:293–299. https://doi.org/10.1016/j.nut.2007.12.007

18. Maes M, Mihaylova I, Leunis JC (2005) In chronic fatigue syndrome, the decreased levels of omega-3 poly-unsaturated fatty acids are related to lowered serum zinc and defects in T cell activation. Neuro Endocrinol Lett 26:745–751

19. Husmann F, Bruhn S, Mittlmeier T, et al (2019) Dietary Nitrate Supplementation Improves Exercise Tolerance by Reducing Muscle Fatigue and Perceptual Responses. Front Physiol 10:404. https://doi.org/10.3389/fphys.2019.00404

20. Zoughaib WS, Hoffman RL, Yates BA, et al (2023) The influence of acute dietary nitrate supplementation on skeletal muscle fatigue and recovery in older women. Physiol Rep 11:e15694. https://doi.org/10.14814/phy2.15694

21. Hadžić E, Starcevic A, Rupčić T, et al (2023) Effects of Soluble Dietary Fibre on Exercise Performance and Perception of Fatigue in Young Basketball Players. Food Technol Biotechnol 61:389–401. https://doi.org/10.17113/ftb.61.03.23.8124

22. Imai A, Oda Y, Ito N, et al (2018) Effects of Dietary Supplementation of Astaxanthin and Sesamin on Daily Fatigue: A Randomized, Double-Blind, Placebo-Controlled, Two-Way Crossover Study. Nutrients 10:. https://doi.org/10.3390/nu10030281

23. Carillon J, Notin C, Schmitt K, et al (2014) Dietary supplementation with a superoxide dismutase-melon concentrate reduces stress, physical and mental fatigue in healthy people: a randomised, double-blind, placebo-controlled trial. Nutrients 6:2348–2359. https://doi.org/10.3390/nu6062348

24. Malaguarnera M, Gargante MP, Cristaldi E, et al (2008) Acetyl L-carnitine (ALC) treatment in elderly patients with fatigue. Arch Gerontol Geriatr 46:181–190. https://doi.org/10.1016/j.archger.2007.03.012

25. Sullivan A, Nord CE, Evengård B (2009) Effect of supplement with lactic-acid producing bacteria on fatigue and physical activity in patients with chronic fatigue syndrome. Nutr J 8:4. https://doi.org/10.1186/1475-2891-8-4
