# Supplementary figures and images for: Vitamin and mineral supplements and fatigue: a prospective study
Source: Eur J Nutr. 2025 Feb 22;64(2):98. doi: 10.1007/s00394-025-03615-y (PMC11889016; doi:10.1007/s00394-025-03615-y)

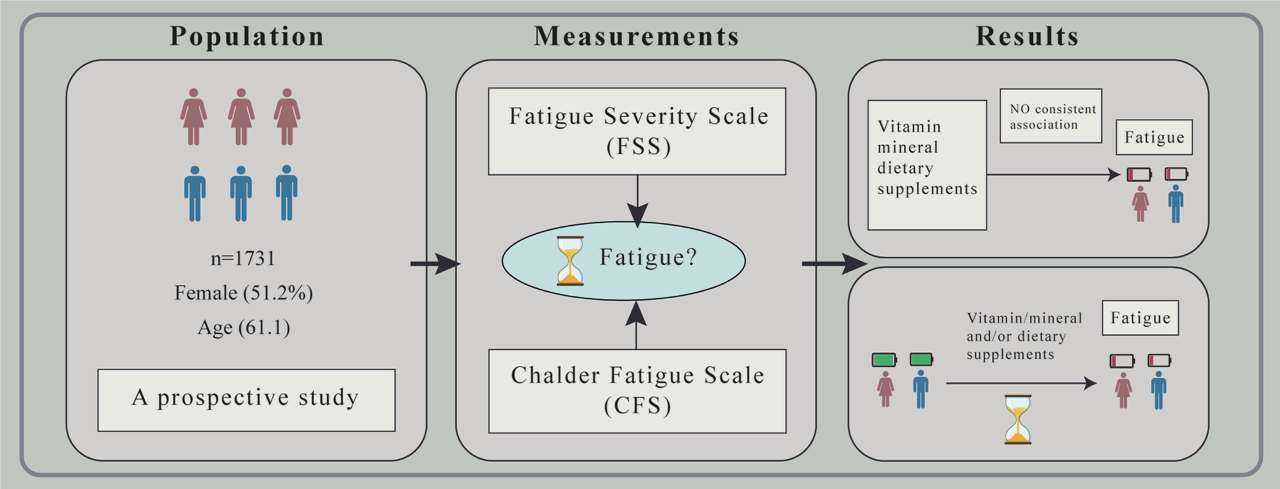

Supplement: Supplementary file 2 — Supplementary Material 2 [file 394_2025_3615_MOESM2_ESM.tiff]
